# Squamous Cell Carcinoma Scalp With Intracranial Extension: Challenging Case of Multidisciplinary Care

**DOI:** 10.7759/cureus.34975

**Published:** 2023-02-14

**Authors:** Ravindran Chirukandath, Sharath k Krishnan, Sumin V Sulaiman, Lijo K Kollannur, Mohammed Nishthar CMT

**Affiliations:** 1 General Surgery, Government Medical College Thrissur, Thrissur, IND; 2 Surgical Oncology, Government Medical College Thrissur, Thrissur, IND; 3 Neurological Surgery, Government Medical College Thrissur, Thrissur, IND; 4 Surgery, Government Medical College Thrissur, Thrissur, IND

**Keywords:** transposition flap, multidisciplinary care, dural involvement, intracranial extension, squamous cell carcinoma

## Abstract

Squamous cell carcinoma (SCC) of the scalp is the second most common non-melanoma cancer of the skin. The incidence of squamous cell carcinoma on the scalp is on the rise, but the intracranial extension is rare. Cranial invasion is rare in SCC of the scalp, but when present, it is associated with a poor prognosis. A 62-year-old female presented with complaints of swelling over the back of her scalp for three months, which rapidly increased in size. She also had a throbbing headache, alopecia in that area, and multiple episodes of pustules in that area. On examination, she had an ulceroproliferative lesion measuring 5*5*3 cm with an irregular surface and varying consistency over the occipital area in the midline surrounded by ulcerations and crusted discharge and fixed to the bone. Contrast-enhanced magnetic resonance imaging (MRI) showed an irregular lesion with the destruction of the right parietal and occipital bones involving both inner and outer tables with intracranial and extracranial components, and the lesion was abutting the superior sagittal sinus. The treatment is surgical resection of the tumor with margin clearance. The treatment plan was designed using a multidisciplinary approach with the collaboration of oncosurgery, neurosurgery, and plastic surgery. The patient underwent wide local excision of the tumor with adequate skin and cranial bone clearance. The tumor was found to have infiltrated the dura mater overlying the superior sagittal sinus. The defect was then closed using a vault prosthetic cover and a scalp transposition flap from the left parietal area. This case report intends to highlight the need for a multidisciplinary approach to the proper management of advanced squamous cell carcinoma to decrease the morbidity and mortality in patients.

## Introduction

Squamous cell carcinoma is the second most common non-melanoma skin cancer, and among malignant scalp tumors, cutaneous SCC is the second most prevalent, preceded by basal cell carcinoma. Due to significant ultraviolet radiation exposure, the head and neck regions are the most affected areas for skin malignancies [1]. The incidence of tumors on the scalp is increasing when compared to those occurring elsewhere on the skin. The incidence of squamous cell carcinoma on the scalp is on the rise, but the intracranial extension is rare [2]. Only 1%-2% of all scalp tumors are malignant; they comprise up to 13% of all malignant cutaneous neoplasms, and cutaneous squamous cell carcinoma is the second most prevalent malignancy [3]. Chiu et al. [4] examined 398 Taiwanese patients with malignant scalp tumors and found that basal cell carcinoma (41.2%) was the most common malignant scalp tumor, followed by squamous cell carcinoma (16.6%). Due to their slow progression, scalp SCC is usually diagnosed before extension into the skull. Invasion into the bone, cortex, and dura mater is rare [5]. But once there is dural or brain invasion, the patient may develop neurological deficits, and the lesion may become inoperable. Dural involvement significantly reduces three-year survival from 83% to 22% [6]. Hence, the depth of invasion is a significant prognostic factor, and cranial involvement is associated with a poorer prognosis [7].

## Case presentation

A 62-year-old female presented with complaints of swelling over the back of the scalp for three months. Swelling rapidly increased in size and was associated with occasional pus discharge. She also had a throbbing headache for the past month. She had a history of alopecia in that area and multiple episodes of pustules in that area since childhood.

**Figure 1:**
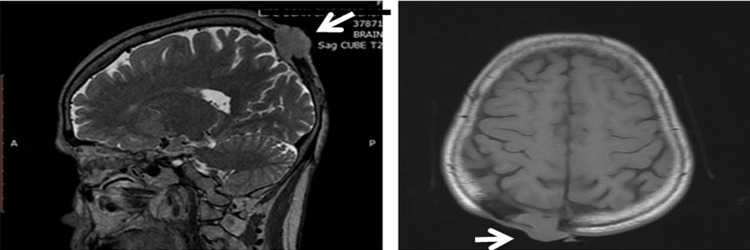
MRI sagittal view An irregular lesion with the destruction of the right parieto-occipital bones involving both inner and outer tables and intracranial extension

On examination, she had an ulceroproliferative lesion measuring 5*5*3 cm with an irregular surface and varying consistency over the occipital area in the midline, surrounded by ulcerations and crusted discharge (Figure 1). The swelling was fixed to the underlying cranial bone, and pus discharge was on expression. There were no neurological deficits. There was no lymph node metastasis and no evidence of distant metastatic disease.

**Figure 2:**
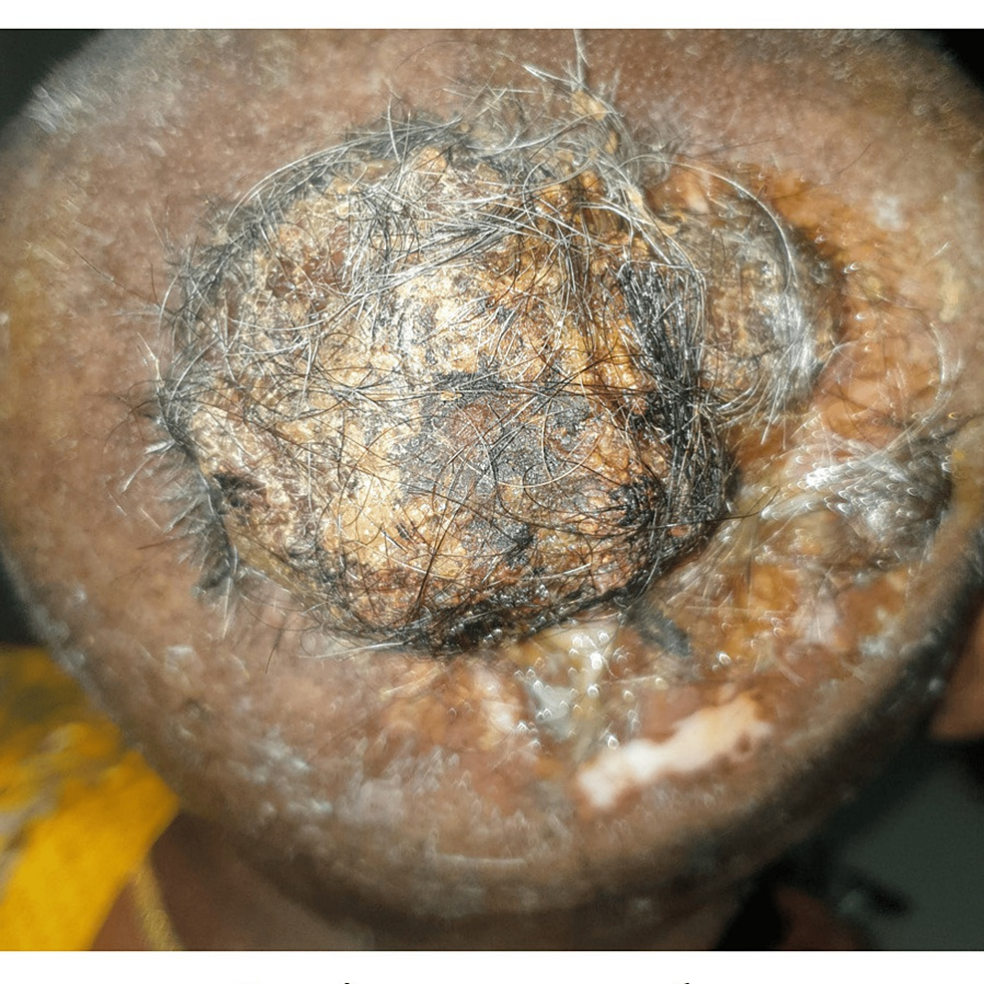
Lesion over the scalp

Contrast-enhanced magnetic resonance imaging showed an irregular lesion with the destruction of the right parieto and occipital bones involving both inner and outer tables with intracranial and extracranial components. The lesion was abutting the superior sagittal sinus with no obvious invasion of brain parenchyma (Figure 2 ).

Wedge biopsy taken from the lesion was reported as well-differentiated squamous cell carcinoma.

**Figure 3:**
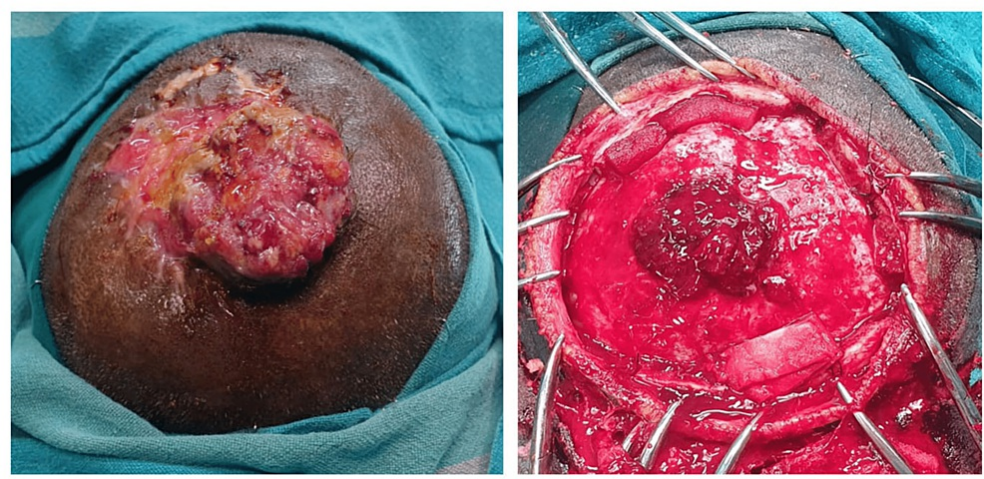
Intraoperative image showing lesion on the scalp and wide local excision of the tumor with skin and bone clearance

The treatment plan was designed using a multidisciplinary approach with the collaboration of oncosurgery, neurosurgery, and plastic surgery. The patient underwent wide local excision of the tumor with adequate skin and cranial bone clearance. The tumor was found to have infiltrated the dura mater overlying the superior sagittal sinus. The defect was then closed by using a scalp transposition flap from the left parietal area (Figure 3).

**Figure 4:**
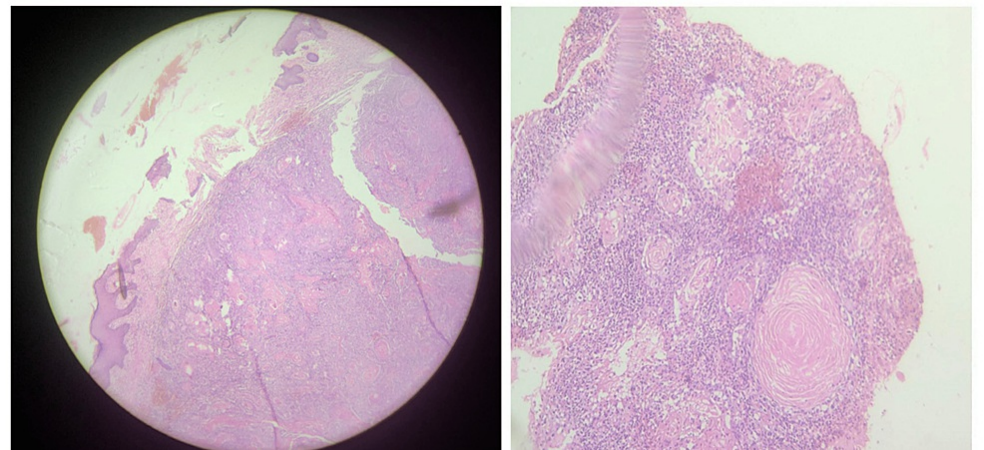
Well-differentiated squamous cell carcinoma involving skull bone and dura mater

Histopathological examination revealed well-differentiated squamous cell carcinoma with a clearance of 2 cm (Figure 4). Skull bone excised was involved by neoplasm with margins free. Tissue adherent to dura was involved by neoplasm.

In the postoperative period, the patient was sent for adjuvant radiotherapy. At a two-month follow-up, her scalp skin defect was well-healed, and she had no neurological deficits.

## Discussion

Cutaneous malignancies of the scalp can be treated successfully with surgical excision, and proper time management is essential as it can prevent local invasion and metastasis. Preoperative imaging is essential to assess the degree of invasion. Computerized tomography is better for the assessment of bone invasion, while MRI is preferred to assess invasion of the dura mater [8].

There are several surgical methods to reconstruct the scalp, such as skin grafts, local tissue transfers, and free flaps. Scalp flap is a safe and reproducible solution for extensive scalp defects and can be performed safely and comfortably with quicker wound healing and cosmetically superior results. The size of the defect, anatomic involvement, contour restoration, hairline maintenance, and return of soft-tissue bulk must all weigh in during the decision-making process. A multidisciplinary approach is essential for the management of tumors with intracranial extension, with assistance from neurosurgeons, plastic surgeons, and radiation oncologists for adjuvant radiotherapy [9].

Cutaneous squamous cell carcinoma is associated with gradual progression, although in rare instances, some tumors show rapid progression. Hence, it is imperative to achieve a sufficient resection margin to prevent local recurrence and metastatic disease.

## Conclusions

Invasive SCC of the scalp with infiltration of bone, dura mater, or brain is a rare entity and very challenging to manage. A thorough preoperative workup and a timely intervention are crucial, as delay can lead to metastasis and inoperability, further increasing the morbidity and mortality risk. A multidisciplinary approach is recommended for the proper management of the disease.
